# Elevated neutrophil-to-lymphocyte ratio and platelet-to-lymphocyte ratio in peripheral blood are associated with higher risk of atherosclerotic renal artery stenosis

**DOI:** 10.3389/fcvm.2026.1737606

**Published:** 2026-02-19

**Authors:** Min Lei, Zhile Li, Hong Ling, Xiaojie Wang, Yuping Wu, Guoqiang Zhong, Ge Xu

**Affiliations:** 1 Department of Cardiology, The First Affiliated Hospital of Guangxi Medical University, Nanning, China; 2 Department of Cardiology, Red Cross Hospital of Yulin City, Yulin, China; 3 Department of Cardiology, Affiliated Hospital of Youjiang Medical University for Nationalities, Baise, China; 4 Laboratory of Atherosclerosis and Ischemic Cardiovascular Diseases, Affiliated Hospital of Youjiang Medical University for Nationalities, Baise, China; 5 Graduate School, Youjiang Medical University for Nationalities, Baise, China; 6 Department of Cardiology, Liuzhou Traditional Chinese Medicine Hospital, Liuzhou, China; 7 Department of Cardiology, The Second Affiliated Hospital of Guangxi Medical University, Nanning, China

**Keywords:** atherosclerotic renal artery stenosis, diagnostic value, neutrophil-to-lymphocyte ratio, platelet-to-lymphocyte ratio, risk factor

## Abstract

**Objectives:**

The prevalence of atherosclerotic renal artery stenosis (ARAS) has been steadily increasing, yet reliable non-invasive diagnostic methods are lacking. This study investigated the association of platelet to lymphocyte ratio (PLR) and neutrophil to lymphocyte ratio (NLR) with atherosclerotic renal artery stenosis (ARAS).

**Methods:**

Data were collected from a total of 1,026 patients, including 362 with ARAS and 664 without. RCS, Logistic regression analysis and stratified analysis were conducted to identify significant risk factors. The diagnostic value of PLR and NLR in predicting the occurrence and severity of ARAS was evaluated using receiver operating characteristic curve analysis.

**Results:**

**NLR** and PLR were elevated in ARAS patients and increased with stenosis severity (NLR r = 0.83). NLR exhibited a nonlinear threshold effect at 3.56, with stronger associations below this cutoff. PLR independently predicted ARAS risk (OR 1.009, *p* < 0.001). ROC analysis showed moderate diagnostic performance, which improved when NLR and PLR were combined with age and serum creatinine (AUC 0.711). In subgroup analyzes, NLR association was amplified in renal impairment, while PLR interaction was significant only in the overweight/obese group.

**Conclusions:**

Elevated levels of NLR and PLR are associated with an increased risk of developing ARAS. Moreover, a higher NLR is linked to more severe stenosis in ARAS cases. Therefore, NLR and PLR have the potential to serve as novel indicators for diagnosing ARAS.

## 1 Introduction

Renal artery stenosis (RAS) is a progressive disease, and a narrowing of one or both renal arteries and their major branches to 50% or more is generally considered clinically significant (1, 2). Atherosclerosis is the main cause of RAS (3, 4). Atherosclerotic renal artery stenosis (ARAS) primarily affects older individuals, especially those with multiple cardiovascular risk factors, while other causes are more prevalent among young people, predominantly in female patients (5, 6). Clinical manifestations of ARAS are nonspecific, making accurate diagnosis challenging and often leading to missed or delayed detection (7). Failure to identify ARAS promptly can result in progressive deterioration of renal function, end-stage renal disease, and refractory hypertension. Early screening, intervention, and treatment are crucial in preventing the development of ARAS into end-stage renal disease (8, 9). Renal artery angiography is the gold standard for accurately evaluating the location, severity, and perfusion of arterial stenosis; however, due to its invasive nature, high cost, procedural risks, and potential complications, it is not routinely employed as a screening method in clinical practice. Therefore, early identification of individuals at risk for ARAS, cautious use of renal arteriography, and timely implementation of therapeutic interventions are of utmost importance.

Mounting evidence from basic and clinical studies suggests that many cardiovascular diseases are chronic inflammation-related conditions involving processes such as endothelial damage, lipid deposition, inflammatory cell aggregation, and vascular remodeling (10–12). Specifically, complete blood count (CBC), is one of the most commonly used, cost-effective and convenient clinical tests. It can reflect the body's inflammatory status, immune response, and changes in blood components. In recent years, CBC has been widely used to assess the risk and prognosis of Atherosclerosis (AS) and its complications. Among CBC parameters, white blood cells and their subsets (neutrophils, lymphocytes), platelets, and derived ratios such as the platelet-to-lymphocyte ratio (PLR) and neutrophil-to-lymphocyte ratio (NLR) have garnered increasing attention in the study of atherosclerosis-related disease pathophysiology and clinical evaluation. These parameters are valued for their ability to reflect the balance between pro-inflammatory and anti-inflammatory and immunoregulatory processes.

Furthermore, in the inflammatory and immunoregulatory response associated with atherosclerosis, neutrophils and platelets act as active participants, promoting endothelial injury, plaque formation, and thrombosis through mechanisms including the release of reactive oxygen species, proteases, chemokines, and neutrophil extracellular trap (NET) formation (13–18). Conversely, lymphocytes are involved in immune regulation, and a decrease in their count has been associated with adverse cardiovascular outcomes. Based on this, NLR and PLR, as integrated inflammatory markers, have been demonstrated to significantly correlate with disease severity, plaque burden, and the risk of adverse events in conditions such as acute myocardial infarction, ischemic stroke, peripheral artery disease, and diabetes-related AS (19–23). The combined use of NLR and PLR can enhance predictive accuracy for cardiovascular events (23).

However, although ARAS is a significant renal manifestation of systemic atherosclerosis and is frequently complicated by cardiac and cerebral atherosclerosis, research on CBC—particularly platelets, neutrophils, PLR, and NLR—in ARAS remains limited in both the number of studies and the depth of mechanistic understanding. Existing literature predominantly focuses on the clinical manifestations, imaging characteristics, and traditional risk factors of ARAS. In contrast, the diagnostic and prognostic value of inflammatory markers from CBC for early identification, risk stratification, and prognostic assessment of ARAS has not yet been systematically elucidated. The primary objective of this study is to explore the correlation between PLR, NLR, and ARAS, analyze the associated risk factors, and provide valuable insights for early clinical detection of ARAS.

## 2 Materials and methods

### 2.1 Study population

A retrospective analysis was conducted on a cohort of 1,026 patients diagnosed with hypertension who underwent renal arteriography at the Department of Cardiovascular Medicine, First Affiliated Hospital of Guangxi Medical University, between January 2020 and December 2022. All cases were suspected to have renal vascular hypertension as the underlying cause of their hypertension. Relevant patient information, including smoking history, diabetes history, medication history and demographic characteristics such as age, sex, height, and weight, was recorded. All patients underwent routine blood tests, renal function assessments, lipid profiles, glycosylated hemoglobin measurements, and renal arteriography. Patients were categorized into the ARAS group (*n* = 362) or the non-ARAS group (*n* = 664) based on the presence or absence of renal artery stenosis.

### 2.2 Inclusion criteria

Patients were included if they met the following criteria: (1) had at least one risk factor for atherosclerosis (e.g., age over 40 years, long-term smoking, diabetes, hyperlipidemia), and met the diagnostic criteria for hypertension according to the 2018 Chinese guidelines for the prevention and treatment of hypertension. (2) provided informed consent and understood the purpose of renal arteriography. (3) complete and accurate clinical data and information.

### 2.3 Exclusion criteria

Patients were excluded if they met any of the following: (1) incomplete clinical data; (2) severe hepatic insufficiency; (3) stage 4 chronic kidney disease (CKD) or higher; (4) dialysis dependence; (5) history of kidney transplantation; (6) malnutrition; (7) acute or chronic infections, malignancies, hematological disorders, tumors, tuberculosis, or other diseases; (8) recent history of acute myocardial infarction or decompensated heart failure; (9) renal stenosis due to non-atherosclerotic causes such as fibromuscular dysplasia or Takayasu arteritis; (10) other forms of secondary hypertension; or (11) previous history of renal artery stent implantation.

### 2.4 Diagnostic criteria

(1) Diagnostic criteria for atherosclerotic renal artery stenosis followed those outlined in the Diagnosis and Management of Renal Artery Stenosis China Expert Consensus (24), including the following: a. Renal artery angiography showing a ≥ 50% decrease in the diameter of the main trunk and/or branch arteries of the renal artery. b. Presence of at least one risk factor for atherosclerosis, such as obesity, diabetes, hyperlipidemia, age over 40 years, or long-term smoking. c. Presence of at least two imaging findings indicative of atherosclerosis, including tapered stenosis or occlusion of the renal artery, eccentric stenosis, irregular plaques, calcification mainly involving the proximal segment and ostium of the renal artery, or manifestations of atherosclerosis in other abdominal blood vessels. (2) Diagnosis of hypertension followed the criteria outlined in the 2018 Chinese Guidelines for the Prevention and Treatment of Hypertension (25), including systolic blood pressure ≥140 mmHg and/or diastolic blood pressure ≥90 mmHg measured on different days or currently taking antihypertensive medication. (3) Diagnosis for diabetes followed the criteria proposed in the Chinese Guidelines for the Prevention and Treatment of Type 2 Diabetes in 2020 (26), including typical diabetes symptoms with elevated blood glucose levels or glycated hemoglobin ≥ 6.5%. (4) Smoking history is defined as continuous smoking of at least one cigarette per day for more than one year, or cessation within the past six months. (5) Medication history was defined as the use of drugs that could affect the results of CBC. This included the long-term use of antiplatelet drugs, statins, angiotensin-converting enzyme inhibitors (ACEIs), and angiotensin receptor blockers (ARBs), as well as the use of steroids and non-steroidal anti-inflammatory drugs within the past two weeks.

### 2.5 Data collection

(1) General Data: Patient information was collected through detailed history-taking and precise measurements. This included sex, age, height, weight, smoking history, diabetes history, medication history and initial standard blood pressure measurement, which was obtained through comprehensive history-taking and precise measurements. The Body Mass Index (BMI) was calculated using recorded height and weight. (2) Laboratory Test Results: Comprehensive documentation of laboratory test results included white blood cell count (WBC), red blood cells (RBC), platelet count (PLT), absolute neutrophil count (NE), absolute lymphocyte count (LYM), monocytes(MONO), hemoglobin(HGB) and red blood cell distribution width (RDW). Additionally, serum creatinine (Scr), cystatin C, uric acid (UA), albumin (Alb), lipid profile (total cholesterol, triglycerides, high-density lipoprotein cholesterol, low-density lipoprotein cholesterol, and lipoprotein a), and glycated hemoglobin (HbA1c) levels were measured. The complete blood count (CBC) is measured using automated hematology analyzers, which count the number of different blood cells (red blood cells, white blood cells, and platelets) in given volume of blood. The platelet-to-lymphocyte ratio (PLR) and neutrophil-to-lymphocyte ratio (NLR) were calculated as PLR = PLT/LYM and NLR = NE/LYM. (3) Renal Arteriography: Percutaneous selective renal arteriography was performed by at least two qualified clinicians to assess the extent of luminal stenosis by measuring the visual diameter. Patients were thoroughly informed about the purpose, potential risks, and precautionary measures associated with renal arteriography, and informed consent was obtained. (4) Group Classification: Based on renal arteriography findings, patients with renal artery stenosis of 50% or more were classified into the ARAS group, while patients with stenosis of less than 50% were classified into the non-ARAS group. Within the ARAS group, further classification was conducted based on stenosis severity into a moderate stenosis group (50% to 70% stenosis) and a severe stenosis group (≥70% stenosis) following the classification criteria recommended by the Society for Cardiovascular Angiography and Interventions in 2014 (27).

### 2.6 Statistical analysis

We assessed the normal distribution of measurement data using the Kolmogorov–Smirnov test. Data that followed a normal distribution was presented as mean ± standard deviation, with differences between groups analyzed using an independent samples *t*-test. For data that did not follow normal distribution, we reported the median and interquartile range, with differences between the two groups evaluated using the Mann–Whitney *U*-test. Categorical data were expressed as frequencies, and differences between groups were compared using the Chi-square test. Spearman's rank correlation analysis was used to assess the correlation between PLR, NLR levels, and ARAS. Logistic regression models were used to identify the risk factors of ARAS, while the ROC curve helped evaluate the predictive value of PLR and NLR for renal artery stenosis. Restricted cubic splines (RCS) were used to investigate the nonlinear associations between biomarkers and ARAS. Piecewise regression models were constructed using generalized linear models (GLMs) to fit segmented regression (two-piece GLMs) within each interval. Stratified analysis was conducted to assess the consistency of associations between PLR, NLR, and ARAS in subgroups. A *p*-value of less than 0.05 was considered statistically significant.

Statistical analysis was conducted using SPSS 27.0 software and R version 4.5.1.

## 3 Results

### 3.1 General information of the ARAS group compared to the non-ARAS group

The ARAS patients exhibited significantly older age, higher systolic blood pressure, and a greater prevalence of medication history compared to the non-ARAS group. (all *p*-values < 0.05). However, there were no significant differences in sex, diastolic blood pressure, or history of smoking and diabetes between the two groups (all *p*-values > 0.05), as shown in Table 1.

**Table 1:** General information of the ARAS group compared to the non-ARAS group.

Variables	ARAS group (*n* = 362)	Non-ARAS group (*n* = 664)	*t*/z/2	*p*
Age (years)	66.00 (58.75, 72.00)	61.00 (54.00, 69.00)	−6.427	*p* < 0.001
Sex (%)			1.334	0.248
Male	226 (62.4)	390 (58.7)		
Female	136 (37.6)	274 (41.3)		
Smoking (%)	152 (42)	242 (36.4)	3.043	0.081
Diabetes (%)	74 (20.4)	162 (24.4)	2.070	0.150
medication (%)	114 (31.5)	285 (42.9)	12.879	*p* < 0.001
Systolic pressure (mmHg)	149 (131, 163)	143 (129, 160)	−2.244	0.025
Diastolic pressure (mmHg)	82 (71, 93)	82 (73, 91)	−0.941	0.347
BMI (kg/m^2^)	24.65 (22.71, 27.61)	24.95 (22.98, 27.63)	−0.075	0.831

### 3.2 Comparison of general information between ARAS moderate stenosis and severe stenosis groups

The ARAS group was further divided based on the degree of stenosis into two categories: The moderate group (50%–70% stenosis, *n* = 104) and the severe stenosis group (≥70% stenosis, *n* = 258). The analysis indicated a significantly higher proportion of patients with diabetes in the ARAS severe stenosis group compared to the moderate stenosis group (*p* < 0.05). However, no statistical differences in terms of age, sex, smoking history, medication history, systolic blood pressure, and diastolic blood pressure between the two groups (*p* > 0.05). Further details can be found in Table 2.

**Table 2:** General information of the ARAS moderate stenosis group compared to severe stenosis group.

Variables	Moderate stenosis group (*n* = 104)	Severe stenosis group (*n* = 258)	*t*/*z*/*χ^2^*	*p* value
Age (years)	66 (59.5, 72)	65 (58, 71.25)	−1.073	0.283
Sex (%)			2.761	0.097
Male	58 (55.8)	168 (65.1)		
Female	46 (44.2)	90 (34.9)		
Smoking (%)	40 (38.5)	112 (43.5)	0.745	0.388
Diabetes (%)	10 (9.6)	64 (24.8)	10.517	0.001
Medication history (%)	34 (32.7)	80 (31.0)	0.097	0.755
Systolic pressure (mmHg)	148.5 (133.25, 170)	149 (131, 162)	−1.281	0.200
Diastolic pressure (mmHg)	79.00 (68.50, 92.00)	83.00 (71.75, 93.25)	−0.780	0.436
BMI (kg/m^2^)	24.57 (22.45, 27.55)	24.65 (22.83, 27.72)	−0.269	0.788

### 3.3 Comparison of test results between ARAS and non-ARAS groups

Comparison of the test results between the ARAS and non-ARAS groups revealed significant differences in several parameters. The ARAS group showed higher levels of white blood cells (WBC), platelets (PLT), neutrophils (NE), triglycerides (TG), serum creatinine (Scr), cystatin C, glycated hemoglobin (HbA1c), platelet-to-lymphocyte ratio (PLR), and neutrophil-to-lymphocyte ratio (NLR) compared to the non-ARAS group. Conversely, lymphocyte count (LYM) and high-density lipoprotein cholesterol (HDL-C) levels were lower in the ARAS group (*p* < 0.05). However, there were no statistically significant differences observed in red blood cells (RBC), hemoglobin(HGB), red blood cell distribution width (RDW), monocytes(MONO), total cholesterol (TC), low-density lipoprotein cholesterol (LDL-C), lipoprotein(a), uric acid(UA) and albumin(Alb) between the ARAS and non-ARAS groups (*p* > 0.05). Further details can be found in the Supplementary Table S1.

### 3.4 Comparison of test results between moderate and severe stenosis groups in ARAS

In ARAS patients, the severe stenosis group showed significantly higher levels of WBC, NE, HbA1c, and NLR compared to the moderate stenosis group (*p* < 0.05). No significant differences were observed in RBC, RDW, LYM, PLT, MONO, HGB, TC, TG, HDL-C, LDL-C, lipoprotein(a), Scr, cystatin C, and PLR between the severe and moderate stenosis groups (*p* > 0.05). Refer to Supplementary Table S2 for details.

### 3.5 Correlation analysis of NLR, PLR, and ARAS

To assess the relationship between PLR, NLR, and ARAS, as well as the degree of stenosis, a correlation analysis was performed. We assigned values to PLR based on its quartiles to convert it into ordinal PLR ≤ 98.18 assigned a value of 0, 98.18 < PLR ≤ 124.75 assigned a value of 1, 124.75 < PLR ≤ 155.51 assigned a value of 2, and PLR > 155.51 assigned a value of 3. We assigned values to NLR based on its quartiles to convert it into ordinal NLR: ≤1.71 assigned a value of 0, 1.71 < NLR ≤ 2.15 assigned a value of 1, 2.15 < NLR ≤ 2.86 assigned a value of 2, and NLR > 2.86 assigned a value of 3. Spearman's rank correlation analysis was conducted. The results indicated that the correlation between PLR and NLR with ARAS was low, with correlation coefficients of 0.188 and 0.174 (both *p* < 0.001); PLR and NLR showed a positive correlation with the degree of ARAS, with correlation coefficients of 0.483 and 0.829 (both *p* < 0.001). See Tables 3, 4.

**Table 3:** Correlation analysis of PLR, NLR and ARAS.

Variables	ARAS	
	R	*p*
PLR	0.188	<0.001
NLR	0.174	<0.001

NLR, neutrophil-to-lymphocyte ratio; PLR, platelet-to-lymphocyte ratio.

**Table 4:** Correlation analysis of PLR, NLR and degree of ARAS stenosis.

Variables	Degree of ARAS stenosis	
	R	*p*
PLR	0.483	<0.001
NLR	0.829	<0.001

NLR, neutrophil-to-lymphocyte ratio; PLR, platelet-to-lymphocyte ratio.

**Figure 1:**
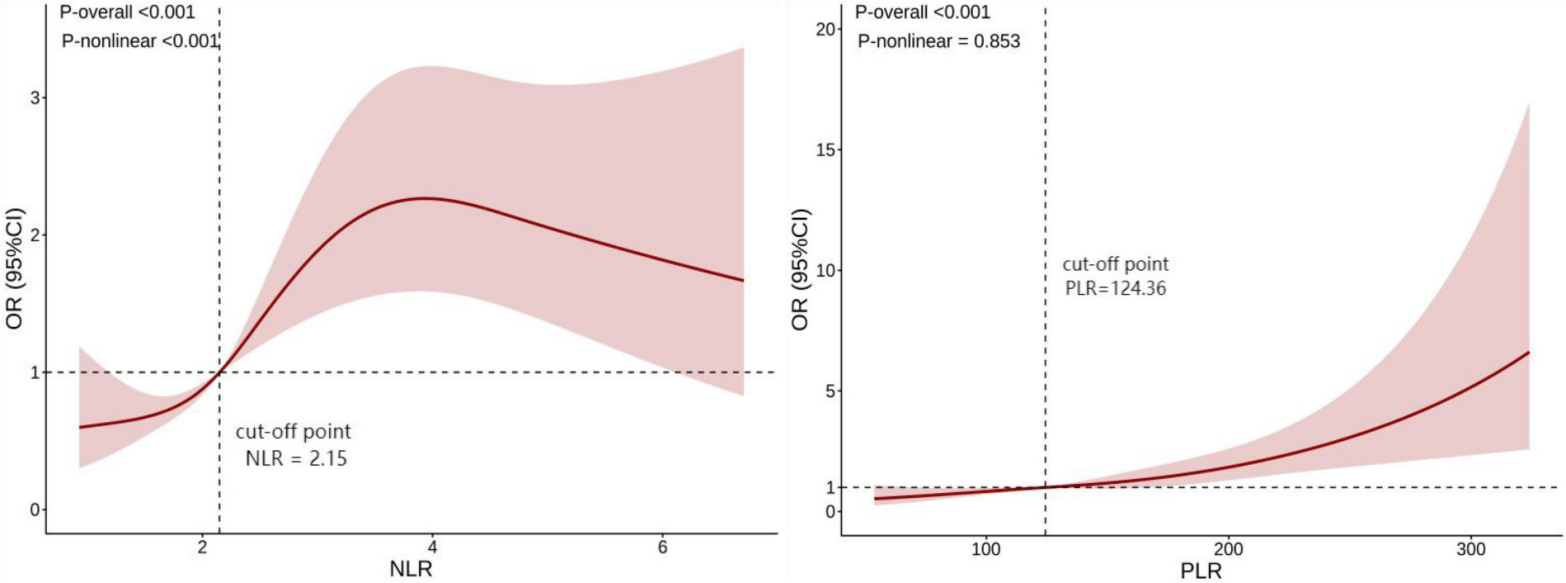
Restricted cubic spline (RCS) analysis of NLR, PLR, and ARAS. Models were adjusted for age, BMI, medication history, systolic pressure, HDL-C, Scr and Cystatin C. (The vertical dashed lines in the figure indicate the optimal cut-off point identified by the model, representing the threshold where the risk association may change). NLR, neutrophil-to-lymphocyte ratio; PLR, platelet-to-lymphocyte ratio; BMI, body mass index; HDL-C, high-density lipoprotein cholesterol; Scr, serum creatinine.

In order to further explore the non-linear relationships between NLR, PLR, and ARAS, we employed restricted cubic splines (RCS) for analysis (Figure 1). There was a linear relationships between PLR and ARAS (*p*-non-linear > 0.05), whereas non-linear relationships were found between NLR and ARAS (*p*-non-linear < 0.05). Then, we further fitted piecewise generalized linear regression models based on the inflection point of NLR. Using an automatic breakpoint detection method, the inflection point of NLR was identified at 3.56. Piecewise regression analysis demonstrated that for NLR values less than or equal to 3.56, each unit increase in NLR significantly increased the risk of ARAS by 63% (OR = 1.63, 95% CI: 1.25–2.13, *p* < 0.001). However, for NLR values greater than 3.56, NLR was not significantly associated with ARAS (OR = 0.80, 95% CI: 0.58–1.09, *p* = 0.151). See Table 5. The log-likelihood ratio test further supported the superiority of the piecewise regression model (likelihood ratio = 12.96, *p* < 0.001).

**Figure 2:**
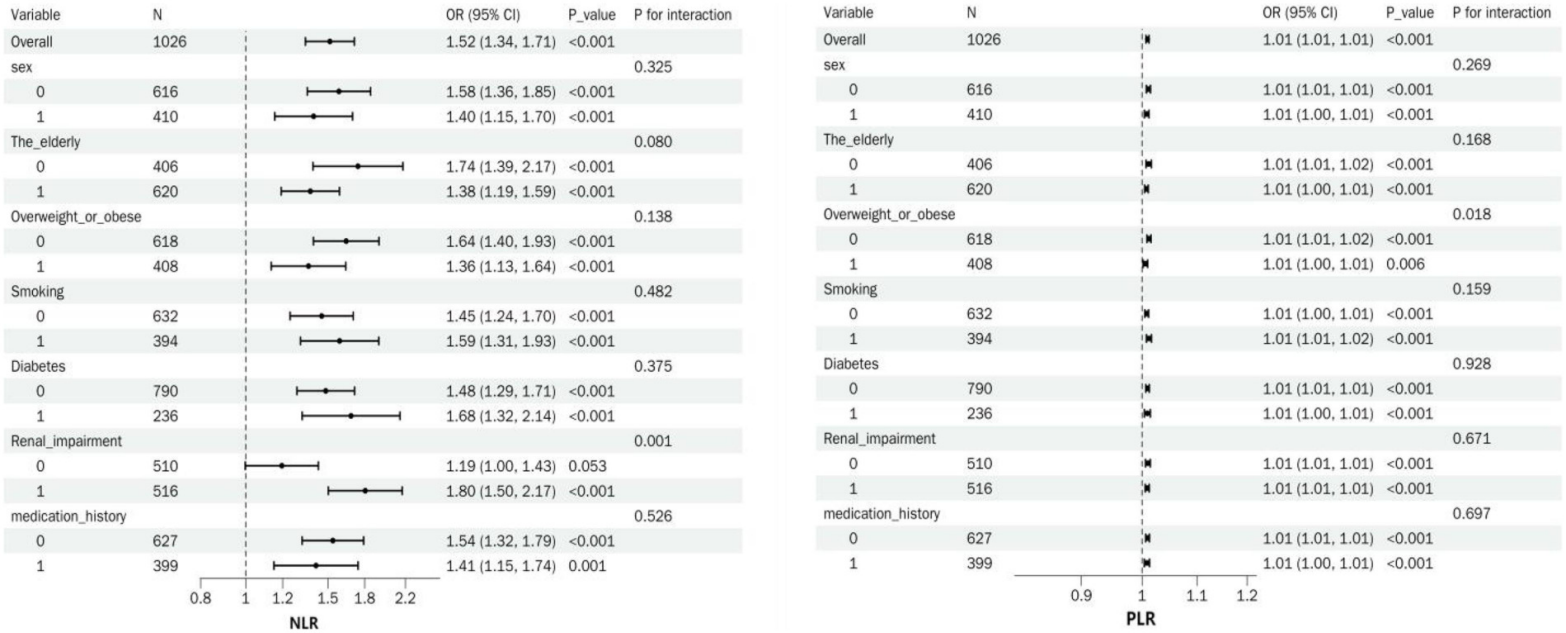
Summary forest plot of NLR, PLR, and ARAS in subgroup analyzes.

**Table 5:** The piecewise generalized linear regression models between NLR and ARAS.

NLR	OR (95% CI)	*p*
Inflection point	3.56	
NLR ≦ 3.56	1.63 (1.25, 2.13)	<0.001
NLR > 3.56	0.80 (0.58, 1.09)	0.151

Models were adjusted for age, BMI, medication history, Systolic pressure, HDL-C, Scr, Cystatin C and PLR.

NLR, neutrophil-to-lymphocyte ratio; PLR, platelet-to-lymphocyte ratio; BMI, body mass index; HDL-C, high-density lipoprotein cholesterol; Scr, serum creatinine.

### 3.6 Univariate logistic regression analysis

To investigate the potential associations between general data, test results, and examination findings of the two groups, univariate logistic regression analysis was performed using the presence of ARAS as the dependent variable. The analysis identified age, BMI, medication history, systolic blood pressure, WBC, PLT, NE, LYM, HDL-C, Scr, cystatin C, PLR, and NLR as risk factors for ARAS (*p* < 0.05). In contrast to the risk factors identified, variables including sex, smoking status, diabetes, diastolic blood pressure, RBC, MONO, HGB, RDW, TC, TG, LDL-C, Lipoprotein a, UA, Alb and HbA1c were not found to be significantly associated with ARAS (*p* > 0.05). Please refer to Supplementary Table S3 for detailed results.

### 3.7 Multivariate logistic regression analysis

To assess potential multicollinearity among the blood cell parameters (WBC, NE, PLT, and LYM), we utilized the collinearity diagnostics function within the linear regression procedure of SPSS statistical software, which calculates the variance inflation factor (VIF) for each variable. It was found that the VIF values for WBC and NE exceeded 10, indicating the presence of multicollinearity. Therefore, WBC and NE were excluded from further analysis. The remaining indicators were examined for covariance. The VIF values for both the NLR and PLR are 1.594, which were below 10, indicating the absence of multicollinearity.

Subsequently, the other significant indicators identified in the univariate logistic regression analysis were included in the multivariate logistic regression analysis to examine the association between PLR, NLR and ARAS (Table 6).

**Table 6:** Multivariate logistic regression analysis of NLR and PLR in relation to ARAS.

Variables	Model 1		Model 2		Model 3	
	OR (95% CI)	*p*	OR (95% CI)	*p*	OR (95% CI)	*p*
NLR	1.280 (1.105–1.483)	<0.001	1.250 (1.076–1.452)	0.003	–	–
PLR	1.006 (1.003–1.009)	<0.001	1.005 (1.002–1.009)	0.001	1.009 (1.007–1.012)	<0.001

Model 1: unadjusted for covariates; Model 2: adjusted for age, BMI, medication history and systolic pressure. Model 3: adjusted for age, BMI, medication history, systolic pressure, HDL-C, Scr and Cystatin C.

The initial unadjusted analysis revealed significant positive associations between PLR, NLR and ARAS (all *p*-values < 0.05). These associations persisted after adjustment for general data in Model 2. However, in the fully adjusted model (Model 3, incorporating laboratory test results), only PLR (OR = 1.009, 95% CI: 1.007–1.012) maintained an independent association with the outcome (*p*-values < 0.05). NLR was not included in the final model.

Subgroup analyzes revealed that NLR and PLR were significantly associated with ARAS across all subgroups (all *p* < 0.05). For NLR, a significant interaction was observed in patients with renal impairment (*p* = 0.001); NLR showed a greater association in those with renal impairment vs. those without (OR = 1.80, 95% CI: 1.50–2.17 vs. OR = 1.19, 95% CI: 1.00–1.43). No other interactions were found related to sex, age, overweight/obesity, smoking, diabetes, or medication history (all *p* > 0.05). In contrast, for PLR, a significant interaction was only noted in the combined overweight/obesity subgroup (*p* = 0.018). No other interactions were significant (all *p* > 0.05) (Figure 2).

Elderly individuals, aged ≥60 years; Overweight or obese, BMI ≥24 kg/m^2^;Renal impairment,Scr >75 μmol/L [This study employed segmented regression models and generalized additive models to analyze the nonlinear relationship between Scr levels and ARAS, adjusting for covariates including age, height, weight, and systolic blood pressure. The results revealed a significant threshold effect, with the optimal breakpoint automatically identified at 75 μmol/L. When Scr was ≤75 μmol/L, each 1 μmol/L increase in Scr was not significantly associated with ARAS (OR = 0.99, 95% CI: 0.98–1.01, *p* = 0.388). However, when Scr was >75 μmol/L, each 1 μmol/L increase in Scr significantly elevated ARAS (OR = 1.02, 95% CI: 1.02–1.03, *p* < 0.001). The difference in odds ratios between these two segments was statistically significant (OR difference = 1.03, 95% CI: 1.01–1.05, *p* = 0.001)].

### 3.8 Characterization of ROC curves for PLR and NLR in predicting ARAS

**Figure 3:**
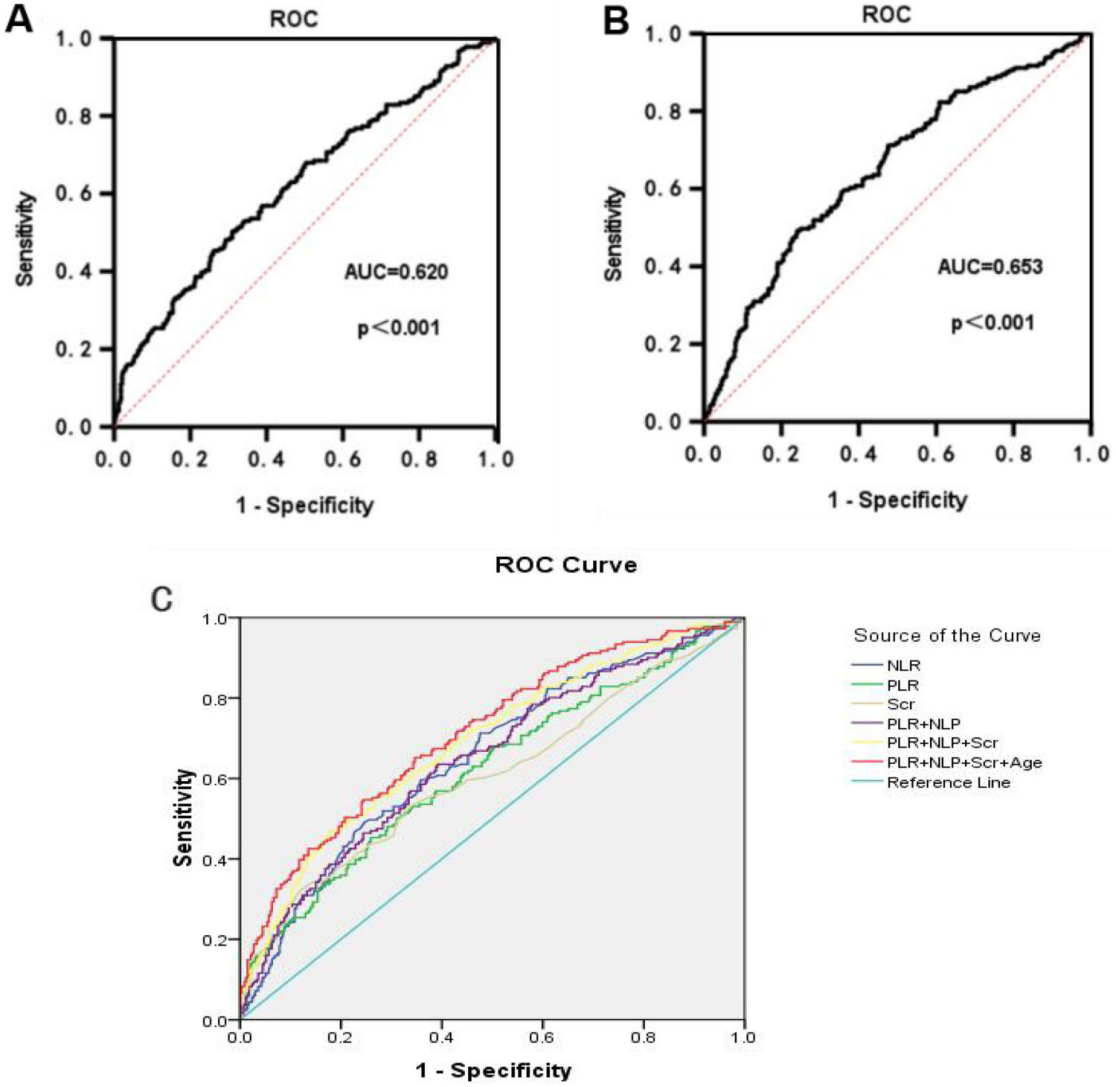
ROC curves for PLR and NLR prediction of ARAS. **(A)** The ROC for PLR in predicting ARAS. **(B)** The ROC for NLR in predicting ARAS. **(C)** The ROC for multi-risk factors in predicting ARAS.

The predictive ability of PLR and NLR for ARAS was assessed using ROC curve analysis. The AUC for PLR in predicting ARAS was 0.620 (95% CI: 0.584 to 0.657), with an optimal diagnostic cut-off value of 145.88. The sensitivity and specificity were determined to be 45.3% and 74.1%, respectively (Figure 3A). Similarly, the ROC curve analysis for NLR in predicting ARAS yielded an AUC of 0.653 (95% CI: 0.617–0.787). The optimal diagnostic cut-off value for NLR was identified as 2.52, yielding a sensitivity of 49.2% and a specificity of 75.9% (Figure 3B). We employed a combination of the PLR, NLR, Scr and age for predictive purposes. This combination significantly improved the predictive capacity for ARAS compared to individual markers. The area under the curve (AUC) for PLR + NLR + Scr + Age in predicting ARAS was 0.711 (95% CI: 0.678 to 0.744). The sensitivity and specificity were determined to be 65.2% and 65.4% (refer to Figure 1C and Table 7 for details).

**Table 7:** ROC curves for prediction of ARAS.

Test Variable(s)	AUC	*p*	Asymptotic 95% confidence interval	Sensitivity	Specificity	Best cutoff value
NLR	0.652	0.000	0.617–0.687	49.20%	75.90%	2.52
PLR	0.62	0.000	0.584–0.656	45.30%	74.10%	145.88
Scr	0.605	0.000	0.568–0.643	32.60%	87.70%	97.5
PLR + NLP	0.649	0.000	0.614–0.684	63.50%	60.80%	–
PLR + NLP + Scr	0.688	0.000	0.654–0.723	47%	82.20%	–
PLR + NLP + Scr + Age	0.711	0.000	0.678–0.744	65.20%	65.40%	–

### 3.9 Characterization of ROC curves for PLR and NLR in predicting ARAS stenosis

**Figure 4:**
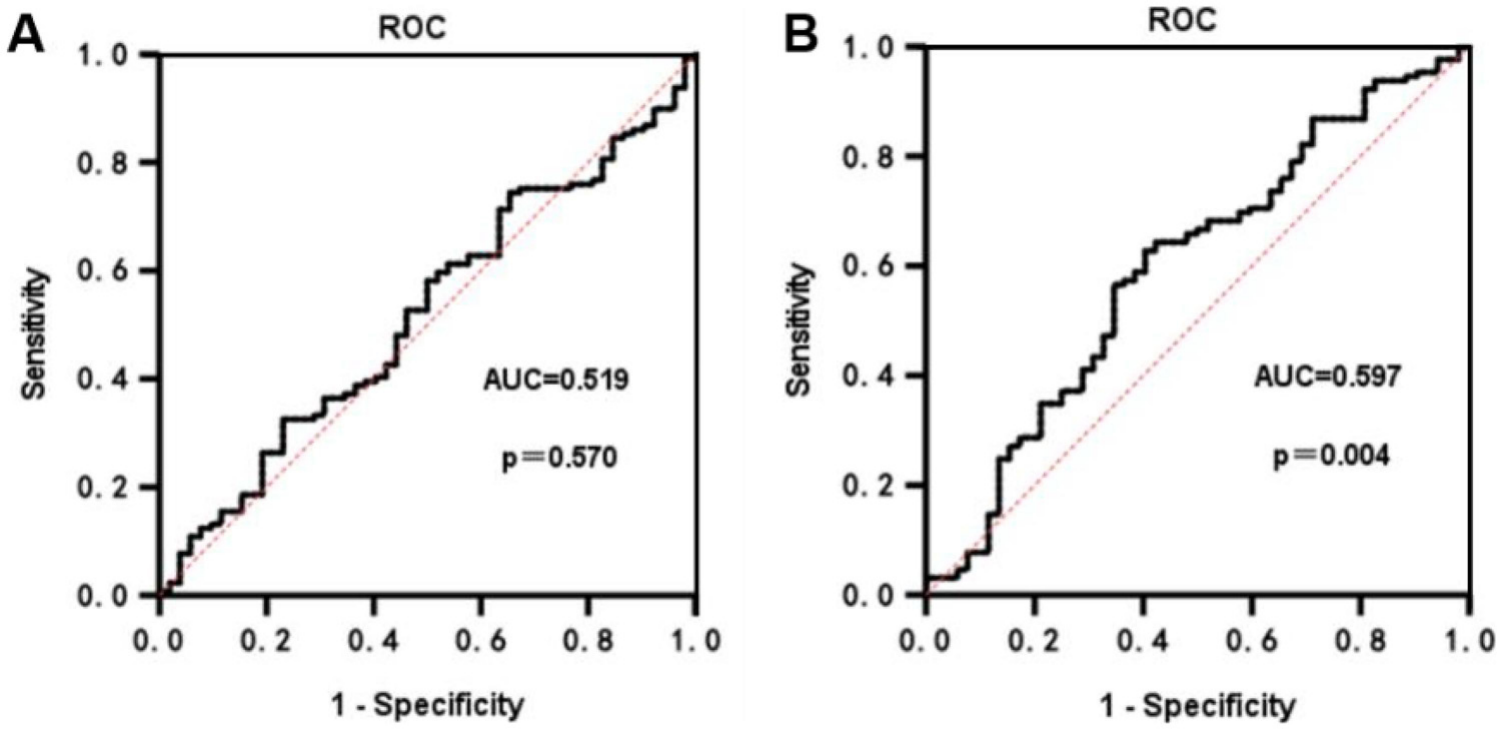
ROC curves for PLR and NLR prediction of ARAS stenosis. **(A)** The ROC for PLR in predicting the degree of ARAS stenosis. **(B)** The ROC for NLR in predicting the degree of ARAS stenosis.

ROC curve analysis was conducted to evaluate the predictive capacity of PLR and NLR for the degree of ARAS stenosis. The AUC for PLR in predicting the degree of ARAS stenosis was found to be 0.519 (*p* = 0.570), indicating that PLR does not provide significant predictive value for determining the degree of ARAS stenosis (Figure 4A). In contrast, the ROC curve for NLR yielded an AUC of 0.597 (95% CI: 0.531–0.663). The optimal diagnostic cut-off value for NLR was determined to be 2.32, with a sensitivity of 62.8% and a specificity of 59.6% (Figure 4B).

## 4 Discussion

Cardiovascular diseases, particularly hypertension, have emerged as significant health concerns globally, largely due to improved living standards and an aging population. ARAS is more frequently observed in patients with resistant hypertension (28) and in patients with widespread atheroma (29). Atherosclerotic renal artery stenosis (ARAS) is a type of renal artery stenosis caused by atherosclerosis. It can lead to several serious health complications. These include secondary hypertension, ischemic nephropathy, renal failure, and congestive heart failure, which may significantly impair overall quality of life (30). The pathogenesis of ARAS is closely linked to hemodynamic changes in the renal artery, whereby renal artery stenosis causes insufficient renal perfusion. This insufficiency, in turn, activates the renin-angiotensin system, further exacerbating hypertension and renal function impairment (31). With continued research into the pathologic mechanism of ARAS, diagnostic and treatment methods have advanced significantly, offering new possibilities for improving patient prognosis (32). However, the insidious onset and lack of specific clinical manifestations make early detection challenging. The diagnosis of ARAS typically relies on imaging examinations, such as Doppler ultrasound, CT angiography, and MRI, which can effectively assess the degree of renal artery stenosis and its impact on renal function (33). Despite the ongoing advancements in imaging technology, the clinical management of ARAS still encounters challenges, particularly in how to select patients suitable for interventional therapy and the risks associated with invasive examinations (9, 32). Therefore, identifying risk factors for ARAS is crucial for early detection and avoiding unnecessary renal arteriography.

This study included 362 patients with ARAS and 664 without ARAS. A comparison of general data revealed that the ARAS group was significantly older with higher systolic blood pressure. Additionally, the proportion of diabetic patients and HbA1c levels was also significantly greater in the severe stenosis group compared to the moderate stenosis group. Advanced age, female participants, diabetes, and hypertension are common risk factors for ARAS and contribute to the progression of renal artery stenosis and various cardiovascular diseases (34). Hypertension is the most prevalent high-risk factor for cardiovascular disease, underscoring the importance of effective blood pressure control. While the role of diabetes as an independent risk factor for ARAS is debated, it did not show relevance in this study.

Triglyceride levels were significantly higher, and HDL-C levels were significantly lower in the ARAS group compared to the non-ARAS group. Hyperlipidemia is a significant risk factor for atherosclerosis and cardiovascular events. Hypertriglyceridemia reflects elevated levels of triglyceride-rich lipoproteins (TRLs) and their remnant cholesterol (RC) in circulation. This remnant cholesterol (RC) is capable of infiltrating the vascular intima, promoting the formation of foam cells, and driving the progression of atherosclerotic plaques (35). HDL-C, known for its protective role against atherosclerosis, promotes reverse cholesterol transport and has anti-inflammatory, anti-oxidant, and endothelial repair properties (36). However, the impact of elevated HDL-C levels on cardiovascular events is still debated. No significant difference in LDL-C and TC levels was observed between the ARAS and non-ARAS groups, possibly due to prior statin therapy among hypertensive patients in both groups.

PLR and NLR, markers of systemic inflammation, were found to be significantly higher in the ARAS group compared to the non-ARAS group. PLR and NLR showed a positive correlation with the occurrence of ARAS, indicating their potential as predictors of the condition. However, their accuracy and sensitivity for predicting ARAS were limited, as indicated by the AUC values. Elevated PLR and NLR levels suggest a higher risk of developing ARAS, while NLR may also contribute to the degree of stenosis. Multivariate logistic regression analysis confirmed PLR and NLR as independent risk factors for ARAS. Subgroup analyzes demonstrated that both NLR and PLR were significantly associated with ARAS across all major demographic and clinical subgroups, further supporting the robustness of these ratios as risk indicators. Notably, the association between NLR and ARAS was significantly stronger in patients with pre-existing renal impairment, indicating a potential synergistic or amplifying effect between NLR and renal dysfunction in predicting ARAS. This observation can be explained by a “vicious cycle” mechanism. Existing renal impairment often comes with chronic low-grade inflammation and immune dysregulation. In this context, a high NLR—indicating increased neutrophil activity and relative lymphopenia—can worsen endothelial injury and oxidative stress, speeding up renal artery atherosclerosis. Moreover, renal impairment may hinder the clearance of inflammatory mediators, intensifying the negative effects of a high NLR. Elevated NLR is consistently associated with increased risk and severity of CKD, correlating with glomerular filtration decline, proteinuria, and mortality (37). In contrast, a significant interaction for PLR was observed only in the overweight/obese subgroup, which may reflect the specificity of PLR in capturing metabolic-inflammatory crosstalk. The combined effects of obesity-related adipokine secretion, chronic low-grade inflammation, and platelet activation likely enhance the indicative value of PLR for atherosclerotic progression in this population. Patients with chronic kidney disease may experience various nutritional and catabolic abnormalities, making the assessment of nutritional status and protein-energy wasting (PEW) crucial in this patient population (38). For all other subgroups—including sex, age, smoking, diabetes, and medication history—no significant interactions with either marker were detected. This suggests that the pro-inflammatory state, as represented by NLR and PLR, and ARAS represents a pervasive risk factor for ARAS, largely independent of traditional demographic characteristics and common comorbidities. Nevertheless, caution is needed in interpreting these results, as limited sample sizes in certain subgroups may have reduced statistical power to detect modest interaction effects.

It is noteworthy that this study found no significant association between RBC, MONO, HGB, RDW, and ARAS. The normal hemoglobin level in ARAS patients suggests that renal ischemia has not yet led to obvious renal anemia. The lack of correlation between RDW and ARAS may reflect that the inflammatory pathway of ARAS has a weak impact on the heterogeneity of erythropoiesis. Alternatively, it may indicate that the sensitivity of RDW is limited in this context. Although monocytes play a central role in atherosclerosis, there was no difference in their absolute peripheral blood counts between groups. This may be because their key role is manifested in local infiltration and activation in the vascular wall rather than changes in circulating levels. In the future (39), it is necessary to further explore the role of monocytes in ARAS by combining monocyte subtype characterization and functional analysis.

Recent ARAS research has focused on novel biomarkers to improve early diagnosis and treatment. Studies show endothelial progenitor cells (EPCs) reflect renal microvascular injury/repair (40), while serum uric acid correlates with long-term prognosis (41). The association of NLR with CKD was stronger in individuals with hypertension (42); however, in our study, no correlation between uric acid and ARAS was observed. This suggests potential differences in the pathophysiological mechanisms underlying these two renal conditions. This study contributes to confirming NLR and PLR as ARAS diagnostic factors, enriching existing biomarkers and suggesting future ARAS biomarkers may not be limited to single biochemical indicators. Routine blood-derived PLR and NLR show promise for predicting ARAS, highlighting the importance of early detection. However, future applications require addressing issues like enhancing predictive accuracy/specificity with clinical indicators, identifying roles of other parameters/inflammation indicators, and exploring whether different inflammatory factor expression patterns predict ARAS, given NLR's link to systemic inflammation.

Although this study provides new insights into the clinical diagnosis and treatment of ARAS, there are still certain limitations. Firstly, this study was retrospective and had limitations due to human and time constraints, which may have introduced bias and affected the final results. Secondly, the study did not follow the patients over a long period, making it difficult to assess the dynamic changes in PLR and NLR and their relationship with ARAS. Consequently, the study did not fully evaluate the impact of ARAS on PLR and NLR. Finally, the study only examined the correlation between some routine blood parameters and ARAS, without including other parameters or common inflammation indicators. Therefore, it was not possible to determine the influence of other routine blood parameters on the study or whether they could be combined with other inflammation indicators to predict the occurrence of ARAS. Further extensive research is needed to explore and confirm these findings.

## 5 Conclusion

This study found that systemic inflammatory markers, PLR and NLR, were significantly elevated in patients with ARAS, and their levels were positively correlated with the degree of renal artery stenosis. Multivariate logistic regression analysis further confirmed that PLR and NLR are independent risk factors for ARAS. Although AUC values indicated limited accuracy of PLR and NLR alone in predicting ARAS was limited, but when combined with clinical indicators such as age and creatinine levels, the sensitivity and specificity of the prediction model were significantly improved. More importantly, PLR and NLR can be conveniently derived from routine blood tests, offering a potentially practical tool for the early screening of ARAS.

However, this study acknowledges certain limitations. As a retrospective analysis, it was subject to sample selection bias and constraints in human and time resources, which may affect the generalizability of the results. Furthermore, the study did not conduct long-term follow-up of patients, nor did it monitor changes in PLR and NLR over time to their association with the ARAS prognosis. As a result, the sustained role of PLR and NLR in disease progression could not be thoroughly evaluated. Future research should focus on optimizing several key areas: integrating imaging omics features with multi-omics biological markers to create a multi-dimensional risk stratification model, validating the stability and predictive efficacy of this model through prospective cohort studies to enhance the specificity and accuracy of predictions; ultimately providing a scientific basis for the early precise identification of ARAS, the development of individualized intervention strategies, and the improvement of prognosis.

## Data Availability

The datasets presented in this article are not readily available because they are internal use only. Requests to access the datasets should be directed to lyzlyg6@sr.gxmu.edu.cn.
