# Supplementary material for: Elevated neutrophil-to-lymphocyte ratio and platelet-to-lymphocyte ratio in peripheral blood are associated with higher risk of atherosclerotic renal artery stenosis
Source: Front Cardiovasc Med. 2026 Feb 19;13:1737606. doi: 10.3389/fcvm.2026.1737606 (PMC12960576; doi:10.3389/fcvm.2026.1737606)
Supplement: Supplementary file 1 [file Datasheet1.docx]

**^Supplementary file^**

**Supplementary table 1. Comparison of test results between the ARAS and non-ARAS groups**

| Variables | ARAS Group（n=362) | Non-ARAS group (n=664) | *t*/*z*/*χ^2^* | *p* |
| --- | --- | --- | --- | --- |
| WBC（*10^9/^L） | 7.30（6.12，8.86） | 6.93（5.84，8.17） | -3.497 | ＜0.001 |
| PLT（*10^9^/L） | 257.00（210.63，294.30） | 237.50（201.13，285.18） | -3.625 | ＜0.001 |
| RBC（*10^12/^L） | 4.60（4.20，5.02） | 4.61（4.23，5.07） | 0.775 | 0.439 |
| NE（*10^9^/L） | 4.48（3.73，5.66） | 4.02（3.24，4.98） | -5.307 | ＜0.001 |
| LYM（*10^9^/L） | 1.79（1.50，2.17） | 1.99（1.64，2.40） | -4.983 | <0.001 |
| MONO（*10^9^/L） | 0.54 (0.44，0.68) | 0.53 (0.44，0.66) | 0.634 | 0.526 |
| HGB(g/L) | 131.80 (121.83，144.00) | 132.35 (123.00，144.63) | -1.159 | 0.247 |
| RDW(%) | 0.13(0.13，0.14) | 0.13（0.13，0.14） | -1.648 | 0.099 |
| TC (mmol/l) | 4.46（3.73，5.46） | 4.59（3.75，5.35） | -0.335 | 0.738 |
| TG (mmol/l) | 1.51（1.06，2.07） | 1.30（0.94，1.91） | -3.913 | ＜0.001 |
| HDL (mmol/l) | 1.03（0.87，1.22） | 1.10（0.92，1.31） | -3.596 | ＜0.001 |
| LDL (mmol/l) | 2.61（2.05，3.29） | 2.63（1.96，3.30） | -1.042 | 0.298 |
| Lipoprotein a (mmol/l) | 0.18（0.08，0.40） | 0.16（0.07，0.42） | -0.371 | 0.710 |
| Scr(umol/l) | 83.00（67.75，105.25） | 73.50（63.00，88.00） | -5.588 | ＜0.001 |
| Cystatin C (mg/L) | 1.02（0.85，1.21） | 0.94（0.83，1.11） | -4.272 | ＜0.001 |
| UA (μmol/L) | 379.00（316.00，458.00） | 374.50（306.00，457.00） | -0.861 | 0.389 |
| Alb (g/L) | 38.60（36.28，41.70） | 38.25（36.13，41.00） | -1.399 | 0.162 |
| HbA1c(%) | 6.40(6.00,7.10) | 6.10（5.80，6.60） | -4.707 | <0.001 |
| NLR | 2.48(1.94,3.33) | 2.02（1.59，2.51） | -8.051 | <0.001 |
| PLR | 140.59(109.75,177.74) | 118.07（94.35，147.77） | -6.362 | ＜0.001 |

Abbreviation: WBC, white blood cells; RBC, red blood cells; PLT, platelets; NE, neutrophils; LYM, lymphocyte; MONO, monocytes; HGB, hemoglobin; RDW, blood cell distribution width; TG, triglycerides; TC, total cholesterol; HDL-C, high-density lipoprotein cholesterol; LDL-C, low-density lipoprotein cholesterol; Scr, serum creatinine; UA, uric acid; Alb, albumin; HbA1c, glycated hemoglobin; NLR, neutrophil-to-lymphocyte ratio; PLR, platelet-to-lymphocyte ratio.

**Supplementary table 2. Comparison of test results between the moderate stenosis group and the severe stenosis group in ARAS**

| Variables | Moderate stenosis group(n=104) | Severe stenosis group(n=258) | *t*/*z*/*χ^2^* | *p* |
| --- | --- | --- | --- | --- |
| WBC（*10^9/^L） | 6.83（5.77，7.90） | 7.6（6.36，9.01） | -3.219 | 0.001 |
| PLT（*10^9^/L） | 252.85（203.57，289.00） | 262.00（212.45，295.23） | -0.801 | 0.423 |
| RBC（*10^12/^L） | 4.62（4.28，5.00） | 4.61（4.20，5.12） | -0.423 | 0.673 |
| NE（*10^9^/L） | 3.98（3.30，5.00） | 4.82（3.81，5.84） | -3.574 | ＜ 0.001 |
| LYM（*10^9^/L） | 1.82（1.56，2.15） | 1.79（1.47，2.19） | -0.033 | 0.973 |
| MONO（*10^9^/L） | 0.52 (0.43，0.71) | 0.55 (0.44，0.66) | -0.789 | 0.431 |
| HGB(g/L) | 131.80 (121.10，145.75) | 132.00 (123.18，144.43) | 0.205 | 0.838 |
| RDW(%) | 0.13(0.13,0.15) | 0.13（0.13，0.14） | -1.298 | 0.194 |
| TC (mmol/l) | 4.45（3.89，5.22） | 4.49（3.69，5.52） | -0.564 | 0.573 |
| TG (mmol/l) | 1.50（1.10，2.07） | 1.55（1.04，2.07） | -0.542 | 0.588 |
| HDL-C(mmol/l) | 1.00（0.90，1.26） | 1.03（0.85，1.22） | -1.037 | 0.300 |
| LDL-C(mmol/l) | 2.61（2.11，3.33） | 2.62（2.03，3.29） | -0.879 | 0.379 |
| Lipoprotein a (mmol/l) | 0.21（0.08，0.36） | 0.16（0.07，0.42） | -1.197 | 0.231 |
| Scr(umol/l) | 81.00（65.00，98.75） | 84.00（65.75，108.00） | -0.830 | 0.406 |
| Cystatin C (mg/L) | 1.04（0.79，1.21） | 1.02（0.86，1.22） | -0.595 | 0.552 |
| UA (μmol/L) | 378.50（305.00，463.00） | 379.50（317.00，458.00） | -0.524 | 0.601 |
| Alb (g/L) | 38.85（35.82，41.80） | 38.55（36.40，41.30） | -0.265 | 0.791 |
| HbA1c(%) | 6.20(5.80,7.13) | 6.40（6.00，7.10） | -1.974 | 0.048 |
| NLR | 2.20(1.79,3.07) | 2.58（2.00，3.47） | -2.895 | 0.004 |
| PLR | 134.82(108.69,166.76) | 141.24（110.88，184.96） | -0.568 | 0.570 |

Abbreviation: WBC, white blood cells; RBC, red blood cells; PLT, platelets; NE, neutrophils; LYM, lymphocyte; PLT, platelets; NE, neutrophils; LYM, lymphocyte; MONO, monocytes; HGB, hemoglobin; RDW, blood cell distribution width; TG, triglycerides; TC, total cholesterol; HDL-C, high-density lipoprotein cholesterol; LDL-C, low-density lipoprotein cholesterol; Scr, serum creatinine; UA, uric acid; Alb, albumin; HbA1c, glycated hemoglobin; NLR, neutrophil-to-lymphocyte ratio; PLR, platelet-to-lymphocyte ratio.

| **Supplementary table 3. Univariate logistic regression analysis** | | | | | | |
| --- | --- | --- | --- | --- | --- | --- |
| Variables | β | SE | Waldχ2 | *p* | OR | 95%CI |
| Age | 0.043 | 0.007 | 39.533 | ＜0.001 | 1.044 | 1.030~1.058 |
| BMI | 0.040 | 0.016 | 6.426 | 0.011 | 1.041 | 1.009~1.074 |
| medication history | 0.492 | 0.138 | 12.782 | ＜0.001 | 1.636 | 1.249~2.143 |
| Systolic pressure | 0.007 | 0.003 | 5.141 | 0.023 | 1.007 | 1.001~1.013 |
| WBC | 0.131 | 0.034 | 15 | ＜0.001 | 1.139 | 1.067~1.217 |
| PLT | 0.004 | 0.001 | 16.133 | ＜0.001 | 1.005 | 1.002~1.007 |
| NE | 0.213 | 0.04 | 27.806 | ＜0.001 | 1.238 | 1.143~1.340 |
| LYM | -0.35 | 0.113 | 9.601 | 0.002 | 0.705 | 0.565~0.879 |
| HDL-C | -0.707 | 0.212 | 11.112 | 0.001 | 0.493 | 0.325~0.747 |
| Scr | 0.016 | 0.002 | 44.694 | ＜0.001 | 1.016 | 1.011~1.020 |
| Cystatin C | 1.219 | 0.231 | 27.914 | ＜0.001 | 3.383 | 2.153~5.318 |
| NLR | 0.416 | 0.062 | 45.341 | ＜0.001 | 1.515 | 1.343~1.710 |
| PLR | 0.009 | 0.001 | 50.157 | ＜0.001 | 1.009 | 1.007~1.012 |
| Sex | 0.155 | 0.134 | 1.333 | 0.248 | 1.167 | 0.898~1.519 |
| Smoking | -0.233 | 0.134 | 3.038 | 0.081 | 0.792 | 0.610~1.029 |
| Diabetes | 0.228 | 0.159 | 2.065 | 0.151 | 1.256 | 0.920~1.714 |
| Diastolic pressure | 0.006 | 0.005 | 1.322 | 0.250 | 1.006 | 0.996~1.016 |
| RBC | 0.003 | 0.003 | 1.757 | 0.185 | 1.003 | 0.998~1.008 |
| MONO | -0.139 | 0.22 | 0.399 | 0.528 | 0.870 | 0.566~1.339 |
| HGB | -0.032 | 0.065 | 0.237 | 0.626 | 0.969 | 0.853~1.101 |
| RDW | 6.469 | 4.48 | 2.085 | 0.149 | 644.995 | 0.099~4197339.787 |
| TC | -0.004 | 0.051 | 0.005 | 0.944 | 0.996 | 0.901~1.102 |
| TG | 0.076 | 0.043 | 3.032 | 0.082 | 1.078 | 0.991~1.174 |
| LDL-C | 0.087 | 0.067 | 1.704 | 0.192 | 1.091 | 0.957~1.244 |
| Lipoprotein a | 0.222 | 0.205 | 1.173 | 0.279 | 1.248 | 0.836~1.864 |
| UA | 0.228 | 0.159 | 2.065 | 0.151 | 1.256 | 0.920~1.714 |
| Alb | 0.015 | 0.017 | 0.741 | 0.389 | 1.015 | 0.981~1.050 |
| HbA1c | 0.028 | 0.026 | 1.167 | 0.28 | 1.028 | 0.978~1.081 |
